# Fruit and Vegetable Intake and the Risk of Chronic Obstructive Pulmonary Disease: A Dose-Response Meta-Analysis of Observational Studies

**DOI:** 10.1155/2020/3783481

**Published:** 2020-02-21

**Authors:** Hongrui Zhai, Yu Wang, Wenjie Jiang

**Affiliations:** Department of Epidemiology and Health Statistics, School of Public Health, Qingdao University, Qingdao, Shandong 266021, China

## Abstract

*Object*. Results on the associations of fruit and vegetable intake with risk of chronic obstructive pulmonary disease (COPD) are still in conflict. Hence, we conducted a meta-analysis to quantitatively evaluate the association between fruit and vegetable intake and the risk of COPD.

**Methods:**

PubMed, Web of Science, and China National Knowledge Infrastructure (CNKI) were searched for relevant studies published up to September 2019. Combined relative risks (RRs) and 95% confidence intervals (CIs) were calculated with the random effects model (REM). Dose-response relationship was assessed by the restricted cubic spline model.

**Results:**

There are 8 studies involving 5,787 COPD cases among 244,154 participants included in this meta-analysis. For the highest versus the lowest level, the pooled RR of COPD was 0.75 (95% CI, 0.68–0.84; *I*^2^ = 46.7%) for fruits plus vegetables, 0.72 (95% CI, 0.66–0.79; *I*^2^ = 46.7%) for fruits plus vegetables, 0.72 (95% CI, 0.66–0.79; *I*^2^ = 46.7%) for fruits plus vegetables, 0.72 (95% CI, 0.66–0.79; *P*_non−linearity_ < 0.01).

**Conclusions:**

This meta-analysis indicates that fruit and vegetable intake might be related to a lower risk of COPD.

## 1. Introduction

Chronic obstructive pulmonary disease (COPD) is a common chronic disease characterized by persistent respiratory symptoms and airflow limitation due to airway inflammation and remodeling [1]. Commonly, airway inflammation and remodeling could promote parenchymal destruction and the development of emphysema, resulting in impaired functional capacity, airway obstruction, and dyspnoea [2]. Furthermore, impaired lung function is associated with an increased risk of serious concomitant diseases [3]. COPD together with its comorbidities causes a heavy economic and social burden around the world, and it is expected to be the third leading cause of death worldwide by 2020 [1]. As mentioned above, COPD is a major public health problem at present.

COPD is a complex disease caused by the interaction of genetic factors with environmental factors, and cigarette smoking is the leading environmental risk factor. However, only 15–20% of ex or current smokers develop COPD during their lifetime [4], suggesting that there are other factors that prevent or promote the development of COPD. Therefore, it is not enough in the prevention of COPD simply by smoking prevention and smoking cessation efforts. Since oxidative stress and inflammation are considered as the main pathogenesis in COPD development and progression [5–7], and fruits and vegetables are foods rich in antioxidants; thus, they have been hypothesized to have a protective effect on COPD. In a 3 yr randomized controlled trial [8], a dietary shift to high fruit and vegetable consumption has been demonstrated to improve lung function in patients with COPD. Some observational studies have been conducted to explore the potential protective effects of fruits and vegetables on the risk of COPD, but the results showed inconsistencies. In terms of fruit intake, reduced risk of COPD was found in four articles [9–12], whereas others revealed no significant relationship [13–15]. In terms of vegetable intake, there are also four articles that showed an inverse association [9, 11, 12, 14]. Given that, we carried out a meta-analysis to evaluate the potential protective effects of fruits and vegetables on COPD, and the dose-response relationship between fruit and vegetable intake and COPD risk.

## 2. Materials and Methods

This meta-analysis was conducted following the guidelines of Meta-analysis Of Observational Studies in Epidemiology (MOOSE) [16].

### 2.1. Search Strategy

Relevant English or Chinese publications were searched in PubMed, Web of Science, and China National Knowledge Infrastructure (CNKI) up to September 2019. The following search strategy was used: (diet OR dietary pattern OR vegetable OR vegetables OR fruit OR fruits) AND (chronic obstructive pulmonary disease OR COPD). The reference lists of retrieved articles were reviewed to identify additional relevant articles. The specific search strategy of PubMed is shown in Supplementary Table S1.

### 2.2. Inclusion Criteria

Study inclusion criteria were as follows: (1) observational studies published in English or Chinese; (2) the exposure of interest was fruit or vegetable intake; (3) the outcome of interest was chronic obstructive pulmonary disease; (4) relative risk (RR), odds ratio (OR) or hazard ratio (HR) with 95% confidence interval (CI) after multivariate adjustment was reported. For dose-response analysis, RR (95% CI) for at least three quantitative categories of fruit or vegetable intake, person-years, and the number of cases for each category of fruit or vegetable intake was provided.

### 2.3. Exclusion Criteria

(1) Review articles, meeting abstracts, and studies written in other languages instead of English or Chinese; (2) unpublished studies; (3) no results reported on the relationship between fruit or vegetable intake and COPD; (4) multivariate adjusted RR and 95% CI not reported, or not possible to calculate; (5) duplicated articles.

Two investigators searched all relevant studies independently. If data from the same population had been published more than once, the most comprehensive one was chosen. If two investigators could not reach an agreement about the eligibility of an article, it was resolved by reaching consensus.

### 2.4. Data Extraction

The following information was extracted from each identified study by two investigators: (1) name of the first author; (2) publication year; (3) continent and country; (4) gender, age range, or mean age of participants; (5) study design; (6) fruit and vegetable intake measurement; (7) measurement method of COPD; (8) sample size; (9) RRs (we presented all results with RR for simplicity) with their 95% CIs for the highest versus lowest level; (10) adjusted covariates. For dose-response analysis, the number of cases, person-years, and the RR (95% CI) for each category of fruit and vegetable intake were extracted. The median or mean level of fruit and vegetable intake for each category was assigned to the corresponding RR for every study. For the unbounded category, we assumed that the boundary had the same amplitude as the adjacent category data [17]. We used servings/day as a standard unit of measurement for fruit and vegetable intake, and a standard portion size of 106 g was used when fruit or vegetable intake was reported by g/day [18].

The Agency for Healthcare Research and Quality (AHRQ) methodology checklist for cross-sectional studies and the Newcastle-Ottawa Scale (NOS) for cohort and case-control studies were used to assess the quality of the included articles [19].

### 2.5. Statistical Analysis

For the pooled measure, we use the inverse variance weighted mean of the logarithm of RRs to calculate the pooled RR with corresponding 95% CIs. Statistical heterogeneity was assessed by the *I*^2^ statistics, *I*^2^ values of 0%, 25%, 50%, and 75% represent no, low, moderate, and high heterogeneity, respectively [20]. Regardless of the value of *I*^2^, random effects model was adopted in all analyses. Random effects model was more conservative than the fixed effects model, and the use of random effects model in all analyses could maintain the consistency of the meta-analysis [21]. Subgroup analysis was conducted by study design, continent where the study conducted, gender, and exposure measures. Metaregression with restricted maximum likelihood estimation was performed to assess the potential covariates that may exert substantial impacts on between-study heterogeneity [22]. Leave-one-out sensitivity analysis was performed if *I*^2^ ≥ 50%, to find which study influences between-study heterogeneity [23]. The influence analysis was conducted by removing one study at a time whether the results would be significantly affected by a single study. The small-study effect was estimated by funnel plot and Egger regression asymmetry test [24]. All statistical analyses were performed using STATA Version 12.0.

For dose-response analysis, a two-stage random effects dose-response meta-analysis [25] was performed. First, a restricted cubic spline model with three knots at the 10th, 50th, and 90th percentiles of levels of fruit and vegetable intake was estimated using generalized least-square regression, taking into account the correlation within each set of published RR. Then the restricted maximum likelihood method was used to combine the study-specific estimates in multivariate random effects meta-analysis [26]. A *P* value is calculated by testing the null hypothesis that the coefficient of the second spline is equal to 0.

## 3. Results

### 3.1. Search Results and Study Characteristics

**Figure 1:**
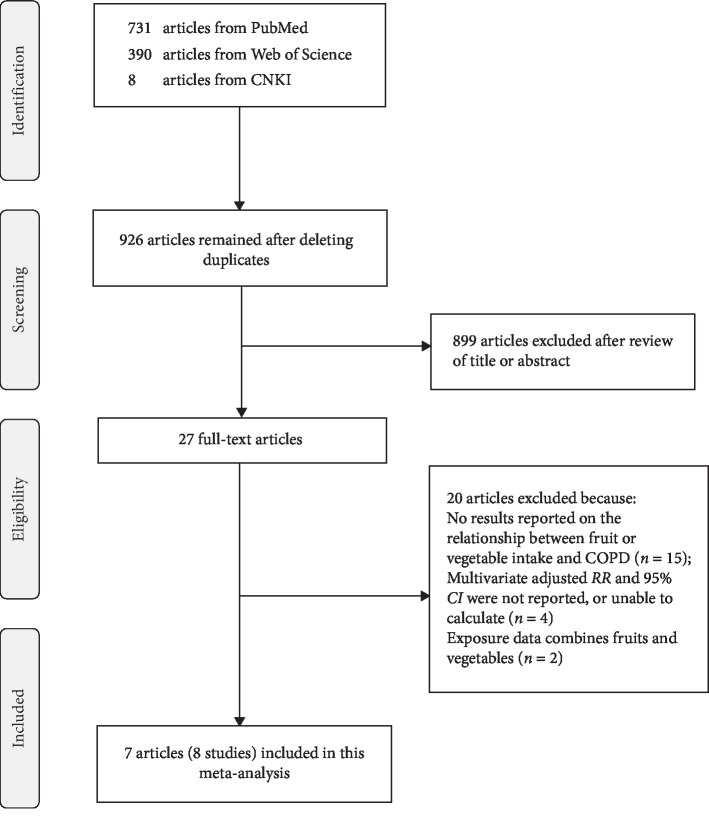
Flow diagram of the literature search for studies.

Literature search identified 731 articles from PubMed, 390 articles from Web of Science, 8 articles from China National Knowledge Infrastructure. A total of 926 articles remained after deleting duplicates. After reviewing the title and abstract, 899 articles were excluded. We further excluded 20 articles after reviewing full-text. At last, a total of seven articles [9–15] (8 studies) were involved in this meta-analysis. The two investigators had no disagreement about the eligibility of all included articles. The flow chart of the literature search is shown in Figure 1. The detailed processes of full-text reviewed articles exclusion are demonstrated in Supplementary Table S2.

Overall, all of these seven articles [9–15] reported results for fruits and vegetables separately. One [13] of the articles contained two independent cohorts with different genders. As a result, eight studies were included, four of which were cohort studies [9, 10, 13], two were case-control studies [14, 15], and two were cross-sectional studies [11, 12].

Contrary to other studies, one study [12] used the highest exposure level as a reference group, so we made a data transformation to use the lowest level as a reference group. In this study [12], vegetable exposure provided two data on raw and cooked vegetables; however, there was no special description on whether the vegetables have been processed in other studies. Considering the local dietary habits of the participants, raw vegetables data were chosen.

In all eight studies, COPD patients were accurately identified by clinical diagnosis. Five studies [12–15] identified COPD patients through physician's diagnosis and spirometric tests; the diagnostic criteria of spirometric tests were FEV_1_/FVC < 0.7; one [12] of these five studies defined airway obstruction as FEV_1_/FVC-ratio below lower limit of normal; *z*-score for FEV_1_/FVC-ratio was −1.96. Two studies [9, 10] identified COPD patients through linkage with the national database and the remaining one [11] by self-reported physician's diagnosis; neither method reported detailed criteria.

For exposure measurement, fruit and vegetable consumption was collected by food frequency questionnaire(FFQ) in six studies [9, 10, 13–15]. Standardized questionnaires were used in one study [11], and a self-designed questionnaire was used in another study [12]. The above questionnaires were designed to collect the frequency of intake of vegetables and fruits over a longer period of time. Specifically, in terms of frequency categories, all types of FFQ [9, 10, 13–15] included eight to nine predefined frequency categories, the self-designed questionnaire [12] included eight categories similarly, and the standardized questionnaire [11] included three categories. Essentially, there was no significant difference in the collection process of exposure information between these seven questionnaires.

The potential confounding factors in the original studies were fully taken into account. As the most important confounding factors, smoking and age were adjusted for in all included studies. Other confounders such as BMI, physical activity, and intake of energy also were adjusted in most studies. The quality assessment showed that the Newcastle-Ottawa score of the cohort studies ranged from seven to nine. For the case-control studies, the Newcastle-Ottawa scores were eight. The quality score of the Agency for Healthcare Research and Quality (AHRQ) methodology checklist ranged from eight to nine for cross-sectional studies. Detailed baseline characteristics of these studies are shown in Table 1. Quality assessment of all included studies is shown in Supplementary Tables S3–S5.

### 3.2. Quantitative Synthesis

The main results are generalized in Table 2.

### 3.3. Fruit plus Vegetable Intake and Risk of COPD

**Figure 2:**
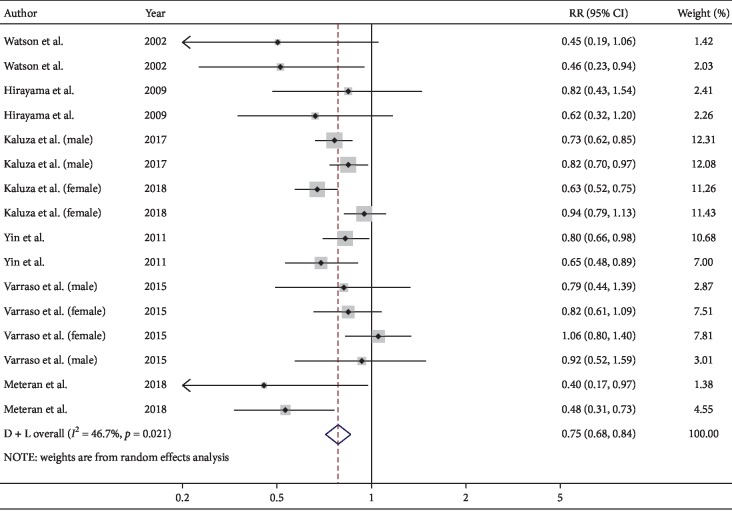
Forest plot for the pooled RR and 95% CI of studies on FV intake and COPD. The size of grey box is positively proportional to the weight assigned to each study, and horizontal lines represent the 95% CIs.

For the highest versus the lowest level of fruit plus vegetable (FV) intake, the pooled RR of COPD was 0.75 (95% CI, 0.68–0.84; *I*^2^ = 46.7%, *P*=0.021; REM; Figure 2). Raw vegetable was included as vegetable intake in one study [12]; the RR (0.77, 95% CI, 0.70–0.85) was consistent with the main result after excluding this article.

The protective effect of FV was observed in all three study designs. No significant results were found in America (RR, 0.92; 95% CI, 0.77–1.10; *I*^2^ = 0%) and female (RR, 0.84; 95% CI, 0.66–1.07; *I*^2^ = 0%). In the subgroup analysis by exposure measures, the beneficial effect of FV intake was found both in FFQ (RR, 0.79; 95% CI, 0.70–0.88; *I*^2^ = 44.1%) and other types of questionnaires (RR, 0.63; 95% CI, 0.48–0.83; *I*^2^ = 54.1%).

### 3.4. Fruit Intake and Risk of COPD

**Figure 3:**
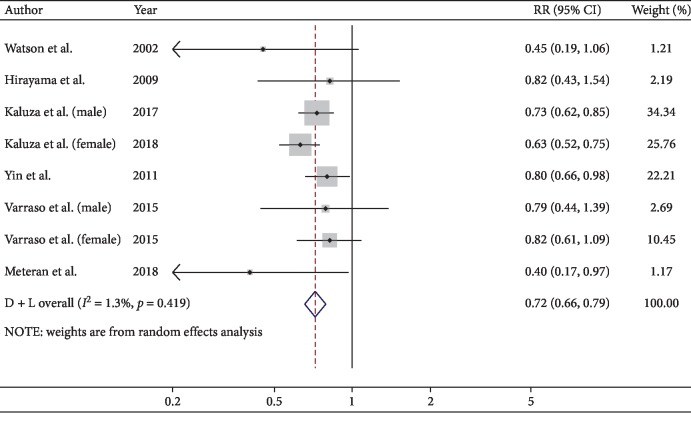
Forest plot for the pooled RR and 95% CI of studies on fruit intake and COPD. The size of grey box is positively proportional to the weight assigned to each study, and horizontal lines represent the 95% CIs.

Data from eight studies including a total of 87,364 participants were used to analyze the association between fruit intake and COPD. For the highest versus the lowest level of fruit intake, the pooled RR was 0.72 (95% CI, 0.66–0.79; *I*^2^ = 1.3%, *P*=0.419; REM; Figure 3). That is, fruit intake might reduce the risk of COPD by 28%.

As for the types of fruits, two studies provided specific fruit types. The inverse association was found in apple or pears (RR, 0.67; 95% CI, 0.55–0.83; *I*^2^ = 0%), while not in banana (RR, 0.87; 95% CI, 0.50–1.51; *I*^2^ = 83.9%) and citrus fruits (RR, 1.05; 95% CI, 0.85–1.28; *I*^2^ = 0%). Three studies reported the RRs of smoking status, the protective effect of fruit was observed in both nonsmokers (RR, 0.78; 95% CI, 0.66–0.93; *I*^2^ = 0%) and ever-smokers (RR, 0.72; 95% CI, 0.64–0.81; *I*^2^ = 17.7%).

Subgroup analyses were conducted by study design, continent where the study conducted, gender, and exposure measures. Fruit intake indicated a beneficial effect on lower the risk of COPD in cohort design (RR, 0.71; 95% CI, 0.63–0.79; *I*^2^ = 0%), while not in cross-sectional design (RR, 0.65; 95% CI, 0.35–1.21; *I*^2^ = 56.8%), and case-control design (RR, 0.66; 95% CI, 0.40–1.11; *I*^2^ = 17.2%). In terms of continent, an inverse association was found in Europe (RR, 0.67; 95% CI, 0.60–0.76; *I*^2^ = 19.6%) and Asia (RR, 0.80; 95% CI, 0.66–0.97; *I*^2^ = 0%), but not in America (RR, 0.81; 95% CI, 0.63–1.05; *I*^2^ = 0%). The beneficial effects of fruit intake were found in male (RR, 0.73; 95% CI, 0.63–0.85; *I*^2^ = 0%), female (RR, 0.70; 95% CI, 0.54–0.90; *I*^2^ = 55.9%) and both genders (RR, 0.76; 95% CI, 0.63–0.91; *I*^2^ = 21.6%). The results used by different types of questionnaires were inconsistent, the beneficial effect was found in FFQ (RR, 0.70; 95% CI, 0.63–0.78; *I*^2^ = 0%), not in other types of questionnaires (RR, 0.65; 95% CI, 0.35–1.21; *I*^2^ = 56.8%).

**Figure 4:**
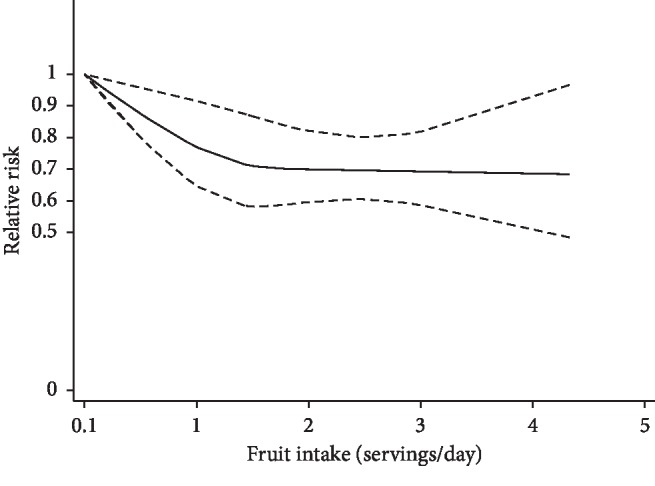
The dose-response analysis of fruit intake and the risk of COPD. The solid line and the long dashed line represent the estimated relative risks and their 95% CIs. The short dashed line represents the linear relationship.

In dose-response analysis, data from four studies [9, 10, 14, 15] were used. Detailed characteristics of studies and participants included in the dose-response analysis are shown in the Supplementary Material Table S6. A nonlinear relationship was found between fruit intake and COPD risk (*P*_non‐linearity_ < 0.01). Compared with the lowest category of fruit intake, the RRs with 95% CIs of COPD risk were 0.78 (95% CI, 0.67–0.92), 0.70 (95% CI, 0.59–0.82), 0.69 (95% CI, 0.59–0.81), and 0.68 (95% CI, 0.48–0.97) for 1, 2, 3, and 4 servings/day, respectively (Figure 4).

### 3.5. Vegetable Intake and Risk of COPD

**Figure 5:**
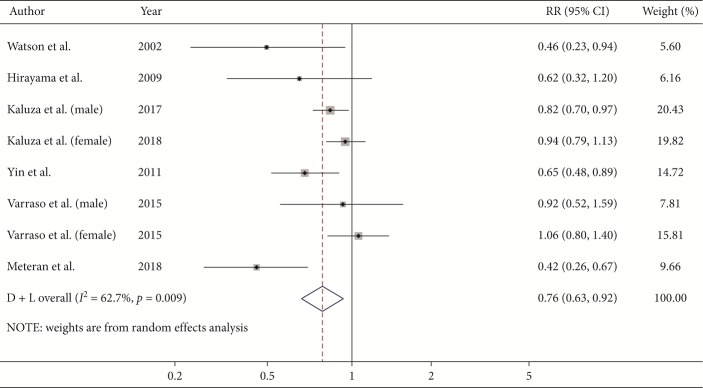
Forest plot for the pooled RR and 95% CI of studies on vegetable intake and COPD. The size of grey box is positively proportional to the weight assigned to each study, and horizontal lines represent the 95% CIs.

Data from eight studies including a total of 246,154 participants were used to analyze the association between vegetable intake and COPD risk. For the highest versus the lowest level of vegetable intake, the pooled RR was 0.76 (95% CI, 0.63–0.92; *I*^2^ = 62.7%, *P*=0.009; Figure 5; REM), which indicates a 24% reduction in the risk of COPD. Since one of the articles [12] was raw vegetables, the pooled result (RR, 0.83; 95% CI, 0.71–0.97) was still consistent with the main result when this article was removed.

In the analysis of vegetable types, two studies provided the specific vegetable types. Green leafy vegetable (RR, 0.75; 95% CI, 0.52–1.09; *I*^2^ = 80.2%), cruciferous vegetable (RR, 1.22; 95% CI, 1.00–1.19; *I*^2^ = 0%), and root vegetable (RR, 1.00; 95% CI, 0.86–1.15; *I*^2^ = 0%) all showed no statistically significant results. Three studies reported the RRs of smoking status; the beneficial effect of vegetable intake was found in ever-smokers (RR, 0.82; 95% CI, 0.72–0.93; *I*^2^ = 0%), but not in nonsmokers (RR, 0.82; 95% CI, 0.53–1.26; *I*^2^ = 63.6%).

In the subgroup analysis by study design, vegetable intake was associated with a lower risk of COPD in case-control design (RR, 0.54; 95% CI, 0.33–0.87; *I*^2^ = 0%) and cross-sectional design (RR, 0.54; 95% CI, 0.36–0.83; *I*^2^ = 56.4%), but not in cohort design (RR, 0.90; 95% CI, 0.81–1.00; *I*^2^ = 0%). In the subgroup analysis by continent, the inverse association was found in Europe (RR, 0.70; 95% CI, 0.53–0.94; *I*^2^ = 76%), and Asia (RR, 0.64; 95% CI, 0.49–0.85; *I*^2^ = 0%), while not in America (RR, 1.03; 95% CI, 0.80–1.32; *I*^2^ = 0%). This protective effect was observed both in male (RR, 0.83; 95% CI, 0.71–0.97; *I*^2^ = 0%) and in both genders (RR, 0.56; 95% CI, 0.45–0.71; *I*^2^ = 0%), but not in female (RR, 0.97; 95% CI, 0.84–1.13; *I*^2^ = 0%). In terms of the questionnaire types, the beneficial effects of vegetable intake were found both in FFQ (RR, 0.88; 95% CI, 0.79–0.98; *I*^2^ = 31.6%) and other types of questionnaires (RR, 0.54; 95% CI, 0.36–0.83; *I*^2^ = 56.4%).

For dose-response analysis of vegetable intake, data from four studies [9, 10, 14, 15] were included. Detailed characteristics of studies and participants included in the dose-response analysis are shown in the Supplementary Material Table S7. A linear trend was found between vegetable intake and COPD risk (*P*_non‐linearity_=0.597), but the results showed no statistical significance.

### 3.6. Metaregression

Moderate heterogeneity (*I*^2^ = 62.7%; *P*=0.009) was found in the analysis of vegetable intake and COPD risk. The metaregression analysis was conducted with the covariates of publication year (*P*=0.290), measurement method of COPD (*P*=0.842), continent (*P*=0.298), exposure measures (*P*=0.052), study design (*P*=0.017), gender (*P*=0.009), and quality score (*P*=0.482) to explore the potential sources of heterogeneity. Study design and gender were found to influence the between-study heterogeneity. We further included study design and gender into the multiple covariates metaregression analysis. The result showed that the estimate of between-study variance Tau-squared was decreased from 0.0331 to 0.

### 3.7. Sensitivity Analysis and Influence Analysis

In the analysis of FV (*I*^2^ = 46.7%; *P*=0.021) and fruit (*I*^2^ = 1.3%; *P*=0.419) intake, low heterogeneity limiting us to perform leave-one-out sensitivity analysis. In vegetable intake and COPD risk, we performed the leave-one-out sensitivity analysis, after excluding one study [12], *I*^2^ was decreased from 62.7% to 43.2% (*P*=0.103), and the result is still significant (RR, 0.83; 95% CI, 0.71–0.97; *I*^2^ = 43.2%, REM). Influence analysis showed that no individual study had an excessive impact on the final results of FV, fruits, and vegetables (Supplementary Figures S1–S3).

### 3.8. Publication Bias

The visual inspection of funnel plot (Supplementary Figures S4–S6) appears to be symmetrical in analysis of FV, fruits, and vegetables. Egger test illustrated that there was no significant publication bias detected in the analysis of FV (*t* = −1.42, *P*=0.178), fruit intake (*t* = −0.65, *P*=0.538) and vegetable intake (*t* = −1.69, *P*=0.142).

## 4. Discussion

In this meta-analysis, seven articles including eight studies were quantitatively summarized to assess the potential protective effect of fruit and vegetable intake on reduction of COPD risk. We found that FV, fruit, and vegetable consumption related to a significantly decreased risk of COPD. In subgroup analysis of FV intake and COPD risk, the inverse association exists in all three study designs. A nonlinear dose-response relationship (*P*_non‐linearity_ < 0.01) was found between fruit intake and COPD risk; the RRs with 95% CIs of COPD risk were 0.78 (95% CI, 0.67–0.92), 0.70 (95% CI, 0.59–0.82) for 1, 2 servings/day of fruits, respectively. For more than 2 servings/day, the risk of COPD was decreased slightly.

The potential mechanism for the protective effect of fruit and vegetable intake on the COPD risk has been proposed as follows. Oxidative stress and inflammation play a major role in the occurrence of COPD [27]. Long-term exposure to tobacco smoke, biomass smoke, and other environmental factors is major causes of oxidative stress in the lung [28–30]. The existence of reactive oxygen species (ROS) impairs endogenous antioxidant defenses [27]. Thus a large amount of ROS may cause proinflammatory gene overexpression and oxidative tissue damage, resulting in inflammation [31]. Fruits and vegetables are rich in vitamin C [32–34], minerals, *β*-carotene [35], dietary fiber [36, 37], and other antioxidants and played a protective role in the development of COPD. It has been observed that dietary antioxidant has the ability to capture exogenous and endogenous free radicals, thereby preventing lipid peroxidation caused by free radicals; for instance, vitamin C can protect *α*_1_-proteinase inhibitors from oxidative damage; therefore, it can maintain the balance between protease and antiprotease [38]. All those antioxidant ingredients can not only ameliorate oxidative stress but also inversely associate with inflammatory biomarkers. Research has shown that vitamins and dietary fibers reduce serum levels of C reactive protein [39].

Between-study heterogeneity is an important part in meta-analysis. Our meta-analysis showed moderate between-study heterogeneity in the analysis of vegetable intake and COPD risk. Study design and gender were found to contribute to the heterogeneity. In multiple covariates metaregression analysis of study design and gender, the estimate of between-study variance Tau-squared was decreased from 0.0331 to 0, which means that study design and gender could explain all sources of heterogeneity [40]. In the leave-one-out sensitivity analysis, one study [12] was identified as a significant impact on the between-study heterogeneity. After excluding that cross-sectional study, heterogeneity was lower than 50% and the result was still statistically significant. In this study [12], cross-sectional survey was performed on two twin study cohorts which included two different twin populations. So it was different from other studies in population selection.

The advantages of our article are in the following aspects. First, a restricted cubic spline model was used in dose-response analysis, and a nonlinear dose-response relationship was found for the COPD risk with fruits. Second, potential confounding factors in original studies were fully taken into account. As the most important confounding factors, smoking and age have been adjusted in all included studies. In addition, BMI, physical activity, intake of energy, and other confounders also have been adjusted in most studies. Third, the quality assessment of included studies was performed, and each of the studies scored more than 7 points, indicating that the articles included were reliable.

There also exist some limitations. First, although results from all 8 studies were adjusted for age, gender, smoking status, and other confounders, the effects of residual or unknown confounding factors on the observed findings could not be ruled out completely. Second, the protective effect of FV was observed in all three study designs, but the results of the three study designs were inconsistent in the analysis of fruits and vegetables. In FV and fruits, inverse association was found in cohort design. Cohort design meets the criteria of temporality and provides stronger evidence for the hypothesis. In the analysis of vegetables, protective effects were found in case-control and cross-sectional design, while cohort design showed an inverse but not significant result. Thus, the protective effects of vegetables on COPD need to be investigated further. Third, the assessment methods of fruit and vegetable consumption were different, which may have a certain impact on the results. In the analysis of fruits and COPD, a significant association was found in studies using FFQ (RR, 0.70; 95% CI, 0.63–0.78), but not in studies using other types of questionnaires (RR, 0.65; 95% CI, 0.35–1.21), so the pooled result (RR, 0.72; 95% CI, 0.66–0.79) was underestimated. In the analysis of vegetables, the pooled result (RR, 0.76; 95% CI, 0.63–0.92) was overestimated by 13.6% due to the use of other questionnaires. Fourth, there were no uniform diagnostic criteria for COPD and no detailed disease information in the included studies. Therefore, the status (Mild, Moderate, Severe) of COPD patients was unclear, and we were unable to perform a pooled analysis.

## 5. Conclusion

The results of this meta-analysis suggest that fruit and vegetable intake may be associated with a reduction of COPD risk. Further research is needed to confirm these results and to explore whether there are gender differences in the protective effects of fruits and vegetables.

## Figures and Tables

**Table 1:** Characteristics of included studies on fruit and vegetable intake and COPD risk.

Author, year [Ref.]	Country	Age range or mean age (gender)	Study design	Sample size (cases)	Exposure measurement	Exposure definition	Outcome assessment	RR (95% CI) for the highest vs. lowest level	Adjustments
Watson et al. 2002 [14]	UK	≥45 (both)	C-C-S	266 (150)	HEA3 Dietary Food Frequency Questionnaire	0–22 VS 220 for fruits (g); 0–49 VS 93 for vegetables (g)	General practitioner's diagnosis and spirometric tests	0.45 (0.19–1.06) for fruits; 0.46 (0.23–0.94) for vegetables	Age, gender, smoking (matched), social class, body mass index, and vegetable intake (when analyzing fruits)
Hirayama et al. 2009 [15]	Japan	50–75 (both)	C-C-S	618 (278)	138-Item food questionnaire	≤141.0 VS ≥ 391.4 for fruits (g/day); ≤110.8 VS ≥ 252.0 for vegetables (g/day)	Physician's diagnosis and spirometric tests	0.82 (0.43–1.54) for fruits; 0.62 (0.32–1.2) for vegetables	Age, gender, BMI (5 years ago), education level (high school or below; college or university), alcohol drinking (nondrinker; drinker), cigarette smoking (never smoker; ex-smoker; current smoker), smoking pack-years, life-long physical activity involvement (never to not any more involved; always been involved), and daily intake of red meat, chicken, and fresh fish
Kaluza et al. 2017 [9]	Sweden	45–79 (male)	C-S	44335 (1918)	FFQ	<0.5 VS ≥ 1.9 for fruits (servings/day)< 1.2 VS ≥ 3.6 for vegetables (servings/day)	Swedish patient register and cause of death register	0.73 (0.62–0.85) for fruits; 0.82 (0.7–0.97) for vegetables	Age (years, continuous), education (less than high school, high school, or university), body mass index (<18.5, 18.5–24.9, 25–29.9 or ≥30 kg/m^2^), total physical activity (MET × hour/day, quintiles), smoking status and pack-years of smoking (never; past <20, 20–39 or ≥40 pack-years; or current < 20, 20–39 or ≥40 pack-years), intake of energy (kcal/day, quintiles), alcohol consumption (g/day, quintiles), and modified recommended food score (scores, continuous) and nonrecommended food score (scores, continuous)
Kaluza et al. 2018 [10]	Sweden	48–83 (female)	C-S	34739 (1512)	FFQ	<0.8 VS ≥ 2.5 for fruits (servings/day); <1.3 VS ≥ 3.1 for vegetables (servings/day)	Swedish Patient Register And Cause Of Death Register	0.63 (0.52–0.75) for fruits; 0.94 (0.79–1.13) for vegetables	Age (years, continuous), education (less than high school, high school or university), BMI (<18.5, 18.5–24.9, 25–29.9 or ≥30 kg/m^2^), total physical activity (MET h/d, quintiles), smoking status and pack-years of smoking (never; past <20, 20–39 or40 pack-years; or current < 20, 20–39 or ≥40 pack-years), dietary supplement use (regular, nonregular or no use), intake of energy (kcal/day, quintiles), alcohol consumption (g/day, quintiles), modified recommended food score (score, continuous) and nonrecommended food score (score, continuous)
Varraso et al. 2015 [13]	USA	40–75 (male)	C-S	47026 (167)	FFQ	Fruit and vegetable score lowest fifth VS highest fifth	Self-reported physician's diagnosis and clinical test	0.79 (0.44–1.39) for fruits; 0.92 (0.52–1.59) for vegetables	Age, physical activity, BMI, total energy intake, smoking status, pack-years of smoking, pack-years of smoking, race/ethnicity, physician visits, US region, and the other AHEI-2010 components
		30–55 (female)	C-S	73228 (723)	FFQ	Fruit and vegetable score lowest fifth VS highest fifth	Self-reported physician's diagnosis and clinical test	0.82 (0.61–1.09) for fruits; 1.06 (0.8–1.4) for vegetables	Age, physical activity, BMI, total energy intake, smoking status, pack-years of smoking, pack-years of smoking, second hand tobacco exposure race/ethnicity, physician visits, US region, spouse's highest educational attainment menopausal status, and the other AHEI-2010 components
Yin et al. 2011 [11]	China	15–69 (both)	C-S-S	32484 (750)	Standardized questionnaire	<2 VS 5–7 for fruits (d/week); <4 VS 6–7 for vegetables (d/week)	Self-reported physician's diagnosis	0.8 (0.66–0.98) for fruit 0.65 (0.48–0.89) for vegetables	Age, gender, urban/rural areas, smoking status, passive smoking exposure and family history
Meteran et al. 2018 [12]	Denmark	58.9 (both)	C-S-S	11458 (289)	Questionnaire	Never VS 1–3 times/week for fruits and raw vegetables	Medical history and the prebronchodilator lung function test	0.4 (0.17–0.97) for fruits; 0.48 (0.31–0.73) for raw vegetables	Age, gender, BMI, smoking, alcohol consumption, and physical activity

COPD: chronic obstructive pulmonary disease; C-C-S: case-control study; C-S: cohort study; C-S-S: cross-sectional study; RR: relative risk; CI: confidence interval; HEA: Health Education Authority in Oxford; FFQ: Food Frequency Questionnaire; AHEI-2010: Alternate Healthy Eating Index 2010. Standardized questionnaire: the frequency of fruit/vegetable intake was divided into three categories according to the number of days normally consumed in a week (<2, 2–4, and 5–7 d/week for fruit; <4, 4-5, 6-7 d/week for vegetables). Questionnaire: based on self-reported frequency of consuming fruits and vegetables on an 8-point scale ranging from 0 to 8 (never to ≥ 4 times/daily) for each category.

**Table 2:** Summary risk estimates of COPD for fruit and vegetable intake by study characteristics.

		No. of studies	Pooled RR	95% CI	*I* ^2^ statistic (%)	*P* value for heterogeneity
Fruits plus vegetables						
	All studies	8	0.75	0.68–0.84	46.7	0.021
	Study design					
	Cohort	4	0.81	0.72–0.92	53.9	0.034
	Cross-sectional	2	0.63	0.48–0.83	54.1	0.088
	Case-control	2	0.59	0.42–0.84	0	0.592
	Continent					
	Europe	4	0.69	0.58–0.82	65.4	0.005
	Asia	2	0.75	0.64–0.88	0	0.653
	America	2	0.92	0.77–1.10	0	0.602
	Gender					
	Both	4	0.64	0.53–0.77	23.3	0.244
	Male	2	0.78	0.70–0.87	0	0.712
	Female	2	0.84	0.66–1.07	77.7	0.004
	Exposure measure					
	FFQ	6	0.79	0.70–0.88	44.1	0.050
	Others	2	0.63	0.48–0.83	54.1	0.088

Fruits						
	All studies	8	0.72	0.66–0.79	1.3	0.419
	Study design					
	Cohort	4	0.71	0.63–0.79	0	0.419
	Cross-sectional	2	0.65	0.35–1.21	56.8	0.128
	Case-control	2	0.66	0.40–1.11	17.2	0.272
	Continent					
	Europe	4	0.67	0.60–0.76	19.6	0.292
	Asia	2	0.80	0.66–0.97	0	0.942
	America	2	0.81	0.63–1.05	0	0.910
	Gender					
	Both	4	0.76	0.63–0.91	21.6	0.281
	Male	2	0.73	0.63–0.85	0	0.795
	Female	2	0.70	0.54–0.90	55.9	0.132
	Exposure measure					
	FFQ	6	0.70	0.63–0.78	0	0.536
	Others	2	0.65	0.35–1.21	56.8	0.128

Vegetables						
	All studies	8	0.76	0.63–0.92	66.7	0.009
	Study design					
	Cohort	4	0.90	0.81–1.00	0	0.424
	Cross-sectional	2	0.54	0.36–0.83	56.4	0.130
	Case-control	2	0.54	0.33–0.87	0	0.545
	Continent					
	Europe	4	0.70	0.53–0.94	76	0.006
	Asia	2	0.64	0.49–0.85	0	0.899
	America	2	1.03	0.80–1.32	0	0.657
	Gender					
	Both	4	0.56	0.45–0.71	0	0.631
	Male	2	0.83	0.71–0.97	0	0.698
	Female	2	0.97	0.84–1.13	0	0.478
	Exposure measure					
	FFQ	6	0.88	0.79–0.98	31.6	0.199
	Others	2	0.54	0.36–0.83	56.4	0.130

COPD: chronic obstructive pulmonary disease; RR: relative risk; CI, confidence interval.

## Supplementary Materials

Supplementary Materials Table S1: the search strategy of fruit and vegetable consumption and COPD in PubMed. Table S2: detailed list of the number of excluded full-text reviewed articles. Table S3: quality assessment of included case-control studies. Table S4: quality assessment of included cross-sectional studies. Table S5: quality assessment of included cohort studies. Table S6: characteristics of studies and participants included in the dose-response analysis of the association between fruit intake and COPD risk. Table S7: characteristics of studies and participants included in the dose-response analysis of the association between vegetable intake and COPD risk. Figure S1: influence analysis of 8 studies on FV consumption and COPD. Figure S2: influence analysis of 8 studies on fruit consumption and COPD. Figure S3: influence analysis of 8 studies on vegetable consumption and COPD. Figure S4: funnel plot of the relative risk of 8 studies on FV consumption and COPD. Each dot represents a different study. Figure S5: funnel plot of the relative risk of 8 studies on fruit consumption and COPD. Each dot represents a different study. Figure S6: funnel plot of the relative risk of 8 studies on vegetable consumption and COPD. Each dot represents a different study. A checklist of MOOSE.
