# Supplementary material for: Fruit and Vegetable Intake and the Risk of Chronic Obstructive Pulmonary Disease: A Dose-Response Meta-Analysis of Observational Studies
Source: Biomed Res Int. 2020 Feb 21;2020:3783481. doi: 10.1155/2020/3783481 (PMC7057001; doi:10.1155/2020/3783481)
Supplement: Supplementary Materials — Table S1: the search strategy of fruit and vegetable consumption and COPD in PubMed. Table S2: detailed list of the number of excluded full-text reviewed articles. Table S3: quality assessment of included case-control studies. Table S4: quality assessment of included cross-sectional studies. Table S5: quality assessment of included cohort studies. Table S6: characteristics of studies and participants included in the dose-response analysis of the association between fruit intake and COPD risk. Table S7: characteristics of studies and participants included in the dose-response analysis of the association between vegetable intake and COPD risk. Figure S1: influence analysis of 8 studies on FV consumption and COPD. Figure S2: influence analysis of 8 studies on fruit consumption and COPD. Figure S3: influence analysis of 8 studies on vegetable consumption and COPD. Figure S4: funnel plot of the relative risk of 8 studies on FV consumption and COPD. Each dot represents a different study. Figure S5: funnel plot of the relative risk of 8 studies on fruit consumption and COPD. Each dot represents a different study. Figure S6: funnel plot of the relative risk of 8 studies on vegetable consumption and COPD. Each dot represents a different study. A checklist of MOOSE. [file 3783481.f1.pdf]

# Supplementary Information

**Table S1** The search strategy of fruit and vegetable consumption and COPD in PubMed.

**Table S2** Detailed list of the number of excluded full-text reviewed articles.

**Table S3** Quality assessment of included case-control studies.

**Table S4** Quality assessment of included cross-sectional studies.

**Table S5** Quality assessment of included cohort studies.

**Table S6** Characteristics of studies and participants included in the dose-response analysis of the association between fruit intake and COPD risk.

**Table S7** Characteristics of studies and participants included in the dose-response analysis of the association between vegetable intake and COPD risk.

**Figure S1** Influence analysis of 8 studies on FV consumption and COPD.

**Figure S2** Influence analysis of 8 studies on fruit consumption and COPD.

**Figure S3** Influence analysis of 8 studies on vegetable consumption and COPD.

**Figure S4** Funnel plot of the relative risk of 8 studies on FV consumption and COPD. Each dot represents a different study.

**Figure S5** Funnel plot of the relative risk of 8 studies on fruit consumption and COPD. Each dot represents a different study.

**Figure S6** Funnel plot of the relative risk of 8 studies on vegetable consumption and COPD. Each dot represents a different study.

Checklist of MOOSE

Table S1: The search strategy of fruit and vegetable consumption and COPD in PubMed.

| Step | Search term (the number of articles) |
|---|---|
| #1 | "chronic obstructive pulmonary disease" OR COPD (82676) |
| #2 | (fruit) OR fruits (163016) |
| #3 | (vegetable) OR vegetables (72489) |
| #4 | (diet) OR “dietary pattern” (502626) |
| #5 | #2 OR #3 OR #4 (667737) |
| #6 | #5 AND #1 (731) |

COPD: chronic obstructive pulmonary diseas.

Table S2: Detailed list of the number of excluded full-text reviewed articles.

| Step | The number of articles before exclusion | Excluding reasons | The number of articles after exclusion | References number of excluded articles |
|---|---|---|---|---|
| 1 | 27 | No results reported on the relationship between fruit or vegetable intake and COPD. | 13 | 1-14 |
| 2 | 13 | Multivariate adjusted *RR* and 95% *CI* were not reported, or unable to calculate. | 9 | 15-18 |
| 3 | 9 | Exposure data conbines fruit and vegetables. | 7 | 19-20 |

COPD: chronic obstructive pulmonary disease; *RR*: relative risk; *CI*: confidence interval.

**References:**


1. Walda I C, Tabak C, Smit H, et al. Diet and 20-year chronic obstructive pulmonary disease mortality in middle-aged men from three European countries. *Eur J Clin Nutr* **2002**, 56, 638-43.

2. Mekary R A. A higher overall diet quality is inversely associated with the risk of chronic obstructive pulmonary disease (COPD) in men and women. *Evid Based Med* **2016**, 21, 36.

3. Butler L M, Koh W P, Lee H P. et al. Prospective study of dietary patterns and persistent cough with phlegm among Chinese Singaporeans. *Am J Respir Crit Care Med* **2006**, 173, 264-270.

4. Ng T P, Niti M, Yap K B, et al. Dietary and supplemental antioxidant and anti-inflammatory nutrient intakes and pulmonary function. *Public Health Nutr* **2014**, 17, 2081-6.

5. Varraso R, Willett WC, Camargo CA Jr. Prospective study of dietary fiber and risk of chronic obstructive pulmonary disease among US women and men. *Am J Epidemiol* **2010**, 171, 776-84.

6. Kaluza J, Harris H, Wallin A, et al. Dietary Fiber Intake and Risk of Chronic Obstructive Pulmonary Disease: A Prospective Cohort Study of Men. *Epidemiology* **2018**, 29, 254-260.

7. Varraso R, S O Shaheen. Could a healthy diet attenuate COPD risk in smokers? *Thorax* **2017**, 72, 491-492.

8. Smit H A. Chronic obstructive pulmonary disease, asthma and protective effects of food intake: from hypothesis to evidence? *Respir Res* **2001**, 2, 261-4.

9. Romieu I C, Trenga. Diet and obstructive lung diseases. *Epidemiol Rev* **2001**, 23, 268-87.

10. Hanson C, Rutten E P. Wouters, E. F. et al. Diet and vitamin D as risk factors for lung impairment and COPD. *Transl Res* **2013**, 162, 219-36.

11. Dastmalchi K, Flores G, Wu S B, et al. Edible Myrciaria vexator fruits: bioactive phenolics for potential COPD therapy. *Bioorg Med Chem* **2012**, 20, 4549-55.

12. Baldrick F R, Elborn J S, Woodside J V, et al. Effect of fruit and vegetable intake on oxidative stress and inflammation in COPD: a randomised controlled trial. *Eur Respir J* **2012**, 39, 1377-84.
13. Hong JY. Effects of dietary antioxidant vitamins on lung functions according to gender and smoking status in Korea: a population-based cross-sectional study. *BMJ Open* **2018**, 8, e020656.
14. Shaheen SO, Jameson KA, Syddall HE, et al. The relationship of dietary patterns with adult lung function and COPD. *Eur Respir J* **2010**, 36, 277-84.
15. Garcia-Larsen V, Potts JF, Omenaas E, et al. Dietary antioxidants and 10-year lung function decline in adults from the ECRHS survey. *Eur Respir J* **2017**, 50.
16. de Batlle J, Romieu I, Antó JM, et al. Dietary habits of firstly admitted Spanish COPD patients. *Respir Med* **2009**, 103, 1904-10.
17. Hanson C, Sayles H, Rutten Eepa, et al. The Association Between Dietary Intake and Phenotypical Characteristics of COPD in the ECLIPSE Cohort. *Chronic Obstr Pulm Dis* **2014**, 1, 115-124.
18. Tabak C, Smit H, A Heederik D, et al. Diet and chronic obstructive pulmonary disease: independent beneficial effects of fruits, whole grains, and alcohol (the MORGEN study). *Clin Exp Allergy* **2001**, 31, 747-55.
19. Patel S, Ram F, Patel SK, et al. Association of behavioral risk factors with self-reported and symptom or measured chronic diseases among adult population (18-69 years) in India: evidence from SAGE study. *BMC Public Health* **2019**, 19, 560.
20. Yang L, Lu X, Deng J, et al. Risk factors shared by COPD and lung cancer and mediation effect of COPD: two center case-control studies. *Am J Respir Crit Care Med* **2015**, 26, 11.

Table S3: Quality assessment of included case-control studies.

| | Author year [ref.] | |
|---|---|---|
| | Watson, L. et al. 2002[14] | Hirayama, F. et al. 2009[15] |
| 1. Selection | | |
| (1) The case definition of osteoporosis is adequate. | * | * |
| (2) Representativeness of the cases. | * | * |
| (3) Selection of controls. | * | * |
| (4) Definition of controls is adequate. | * | * |
| 2. Comparability | | |
| (1) Comparability of cases and controls on the basis of the design or analysis | ** | ** |
| 3. Exposure. | | |
| (1) Ascertainment of Exposure. | * | * |
| (2) Same method of ascertainment for cases and controls | * | * |
| (3) Non-Response Rate. | No description | No description |
| Overall quality score | 8 | 8 |

The quality of studies was assessed by the Newcastle-Ottawa quality assessment scale.
One star represents a score of 1, and a study can be awarded a maximum score of 9 (9 stars) in total.

Table S4: Quality assessment of included cross-sectional studies.

| | Author yea[ref.] | |
|---|---|---|
| | Yin, P. et al. 2011[11] | Meteran, H. et al. 2018[12] |
| 1. Define the source of information (survey, record review); | 1 | 1 |
| 2. List inclusion and exclusion criteria for exposed and unexposed subjects (cases and controls) or refer to previous publications; | 1 | 1 |
| 3. Indicate time period used for identifying patients; | 1 | 1 |
| 4. Indicate whether or not subjects were consecutive if not population-based; | 1 | 1 |
| 5. Evaluators of subjective components of study were not masked to other aspects of the status of the participants; | 1 | 0 |
| 6. Describe any assessments undertaken for quality assurance purposes (e.g., test/retest of primary outcome measurements); | 1 | 1 |
| 7. Explain any patient exclusions from analysis; | 0 | 1 |
| 8. Describe how confounding was assessed and/or controlled; | 1 | 1 |
| 9. If applicable, explain how missing data were handled in the analysis | 0 | 0 |
| 10. Summarize patient response rates and completeness of data collection; | 0 | 1 |
| 11. Clarify what follow-up, if any, was expected and the percentage of patients for which incomplete data or follow-up was obtained; | 1 | 1 |
| Overall quality score | 8 | 9 |

The quality of studies was assessed by the Agency for Healthcare Research and Quality (ARHQ) methodology checklist. 1= “Yes”, 0= “No” or “Unclear”. The full score for the scale is 11 points.

Table S5: Quality assessment of included cohort studies.

| First author (year) [ref.] | Representativeness of the exposed cohort | Selection of the unexposed cohort | Ascertainment of exposure | Outcome of interest not present at start of study | Control for important factor or additional factor† | Outcome assessment | Follow-up long enough for outcomes to occur‡ | Adequacy of follow-up of cohorts§ | Overall quality score |
|---|---|---|---|---|---|---|---|---|---|
| Kaluza, J et al. (2017) [9] | * | * | * | * | ** | * | * | * | 9 |
| Kaluza, J et al. (2018) [10] | * | * | * | * | ** | * | * | * | 9 |
| Varraso, R et al. (2015) [13] | | * | * | * | ** | * | * | | 7 |

A study could be awarded a maximum of one star for each item except for the item Control for important factor or additional factor.

† A maximum of 2 stars could be awarded for this item. Studies that controlled for age or gender received one star, whereas studies that controlled for other important confounders such as chronic health conditions received an additional star.

‡ A cohort study with a follow-up time >3 years was assigned one star.

§ A cohort study with a follow-up rate >70% was assigned one star.

Table S6: Characteristics of studies and participants included in the dose-response analysis of the association between fruit intake and COPD risk.

| Author (Year) | Study design | Dose (Servings/day) | *RR*(95%*CI*) | Person years | Cases | Gender | Adjustment for covariant |
|---|---|---|---|---|---|---|---|
| Watson, L. (2002) | C-C-S | 0.1 | 1 | 94 | 57 | Both | Smoking-matched; adjusted for age, gender, body mass index and vegetable intake(when analyzing fruit). |
| | | 0.67 | 0.97(0.5-1.85) | 125 | 76 | | |
| | | 1.6 | 0.45(0.19-1.06) | 47 | 17 | | |
| Hirayama, F. (2009) | C-C-S | 0.67 | 1 | 181 | 97 | Both | Age, gender, BMI (5 years ago), education level (high school or below; college or university), alcohol drinking (non-drinker; drinker), cigarette smoking (never smoker; ex-smoker; current smoker), smoking pack-years, life-long physical activity involvement (never to not any more involved; always been involved), and daily intake of red meat, chicken and fresh fish |
| | | 1.87 | 0.57(0.32-1.03) | 151 | 67 | | |
| | | 3.05 | 0.63(0.34-1.17) | 107 | 53 | | |
| | | 4.34 | 0.82(0.43-1.54) | 143 | 60 | | |
| Kaluza, J. (2017) | C-S | 0.3 | 1 | 103166 | 536 | Male | Age (years, continuous), education (less than high school, high school or university), body mass index (<18.5, 18.5–24.9, 25–29.9 or≥30 kg/m2), total physical activity (MET×hour/day, quintiles), smoking status and pack-years of smoking (never; past <20, 20–39 or ≥40 pack-years; or current <20, 20–39 or ≥40 pack-years), intake of energy (kcal/day,quintiles), alcohol consumption (g/day, quintiles) and modified recommended food score (scores, continuous) and non-recommended food score (scores, continuous). |
| | | 0.6 | 0.9(0.79-1.03) | 124777 | 417 | | |
| | | 1 | 0.85(0.73-0.97) | 117483 | 344 | | |
| | | 1.5 | 0.88(0.76-1.02) | 119185 | 338 | | |
| | | 2.5 | 0.73(0.62-0.85) | 119335 | 283 | | |
| Kaluza, J. (2018) | C-S | 0.6 | 1 | 78467 | 546 | Female | Age (years, continuous), education (less than high school, high school or university), BMI (<18.5, 18.5–24.9, 25–29.9 or≥30 kg/m2), total physical activity (MET h/d, quintiles), smoking status and pack-years of smoking (never; past <20, 20–39 or40 pack-years; or current <20, 20–39 or ≥40 packyears), dietary supplement use (regular, non-regular or no use), intake of energy (kcal/day, quintiles), alcohol consumption (g/day, quintiles), modified Recommended Food Score (score, continuous) and Non-Recommended Food Score (score, continuous). |
| | | 1.1 | 0.8(0.69-0.93) | 80012 | 305 | | |
| | | 1.5 | 0.73(0.62-0.86) | 80871 | 245 | | |
| | | 2 | 0.78(0.66-0.92) | 81943 | 234 | | |
| | | 2.9 | 0.63(0.52-0.75) | 81152 | 181 | | |

The number of person years in case-control studies(C-C-S) was the total of participants in each category. *RR*, relative risk; *CI*, confidence interval; C-C-S, case-control study; C-S, cohort study.

Table S7: Characteristics of studies and participants included in the dose-response analysis of the association between vegetable intake and COPD risk.

| Author (Year) | Study design | Dose (Servings/day) | *RR*(95%*CI*) | Person years | Cases | Gender | Adjustment for covariant |
|---|---|---|---|---|---|---|---|
| Watson, L. (2002) | C-C-S | 0.23 | 1 | 88 | 57 | Both | Smoking-matched; adjusted for age, gender, body mass index and vegetable intake(when analyzing fruit). |
| | | 0.67 | 0.74(0.36-1.51) | 88 | 51 | | |
| | | 1.08 | 0.46(0.23-0.94) | 88 | 40 | | |
| Hirayama, F. (2009) | C-C-S | 0.52 | 1 | 188 | 103 | Both | Age, gender, BMI (5 years ago), education level (high school or below; college or university), alcohol drinking (non-drinker; drinker), cigarette smoking (never smoker; ex-smoker; current smoker), smoking pack-years, life-long physical activity involvement (never to not any more involved; always been involved), and daily intake of red meat, chicken and fresh fish |
| | | 1.3 | 0.67(0.37-1.19) | 156 | 71 | | |
| | | 1.96 | 0.71(0.39-1.28) | 144 | 59 | | |
| | | 2.79 | 0.62(0.32-1.2) | 129 | 44 | | |
| Kaluza, J. (2017) | C-S | 0.8 | 1 | 110402 | 578 | Male | Age (years, continuous), education (less than high school, high school or university), body mass index (<18.5, 18.5–24.9, 25–29.9 or≥30 kg/m2), total physical activity (MET×hour/day, quintiles), smoking status and pack-years of smoking (never; past <20, 20–39 or ≥40 pack-years; or current <20, 20–39 or ≥40 pack-years), intake of energy (kcal/day,quintiles), alcohol consumption (g/day, quintiles) and modified recommended food score (scores, continuous) and non-recommended food score (scores, continuous). |
| | | 1.6 | 0.95(0.83-1.08) | 1168466 | 418 | | |
| | | 2.2 | 0.98(0.85-1.13) | 118041 | 368 | | |
| | | 3 | 0.89(0.77-1.04) | 119978 | 299 | | |
| | | 4.6 | 0.82(0.70-0.97) | 118698 | 255 | | |
| Kaluza, J. (2018) | C-S | 0.9 | 1 | 75985 | 453 | Female | Age (years, continuous), education (less than high school, high school or university), BMI (<18.5, 18.5–24.9, 25–29.9 or≥30 kg/m2), total physical activity (MET h/d, quintiles), smoking status and pack-years of smoking (never; past <20, 20–39 or40 pack-years; or current <20, 20–39 or ≥40 packyears), dietary supplement use (regular, non-regular or no use), intake of energy (kcal/day, quintiles), alcohol consumption (g/day, quintiles), modified Recommended Food Score (score, continuous) and Non-Recommended Food Score (score, continuous). |
| | | 1.5 | 0.85(0.73-0.99) | 80667 | 296 | | |
| | | 2 | 0.95(0.81-1.11) | 81517 | 277 | | |
| | | 2.6 | 0.88(0.74-1.04) | 82116 | 243 | | |
| | | 3.7 | 0.94(0.79-1.13) | 82159 | 243 | | |

The number of person years in case-control studies(C-C-S) was the total of participants in each category. *RR*, relative risk; *CI*, confidence interval; C-C-S, case-control study; C-S, cohort study.

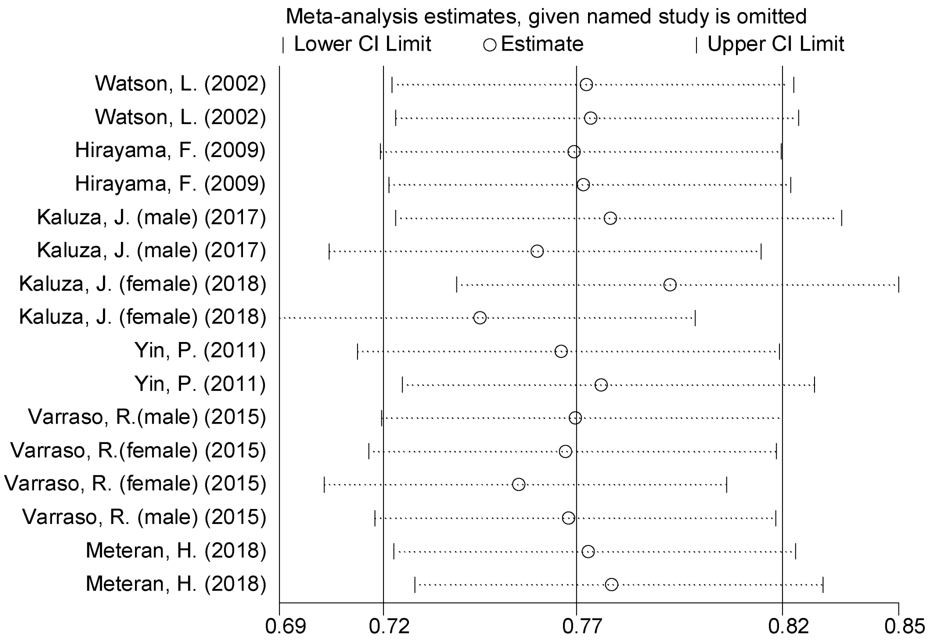


Figure S1: Influence analysis of 8 studies on FV consumption and COPD.

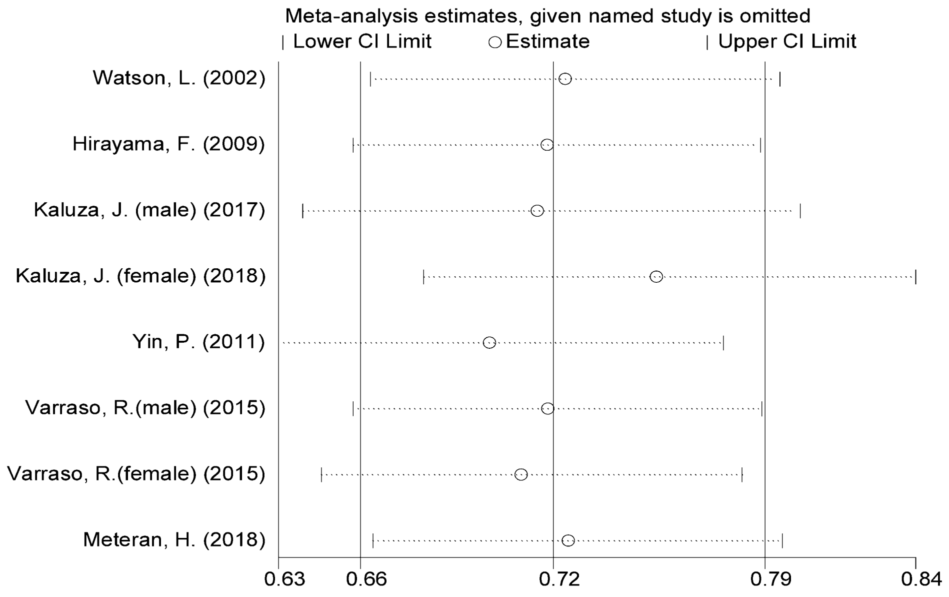


Figure S2: Influence analysis of 8 studies on fruit consumption and COPD.

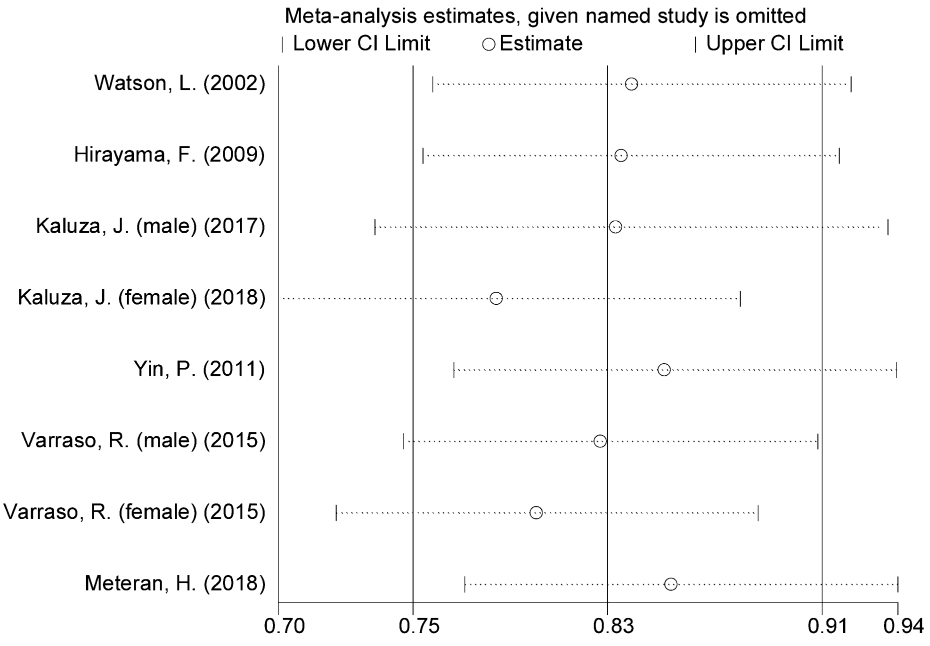


Figure S3: Influence analysis of 8 studies on vegetable consumption and COPD.

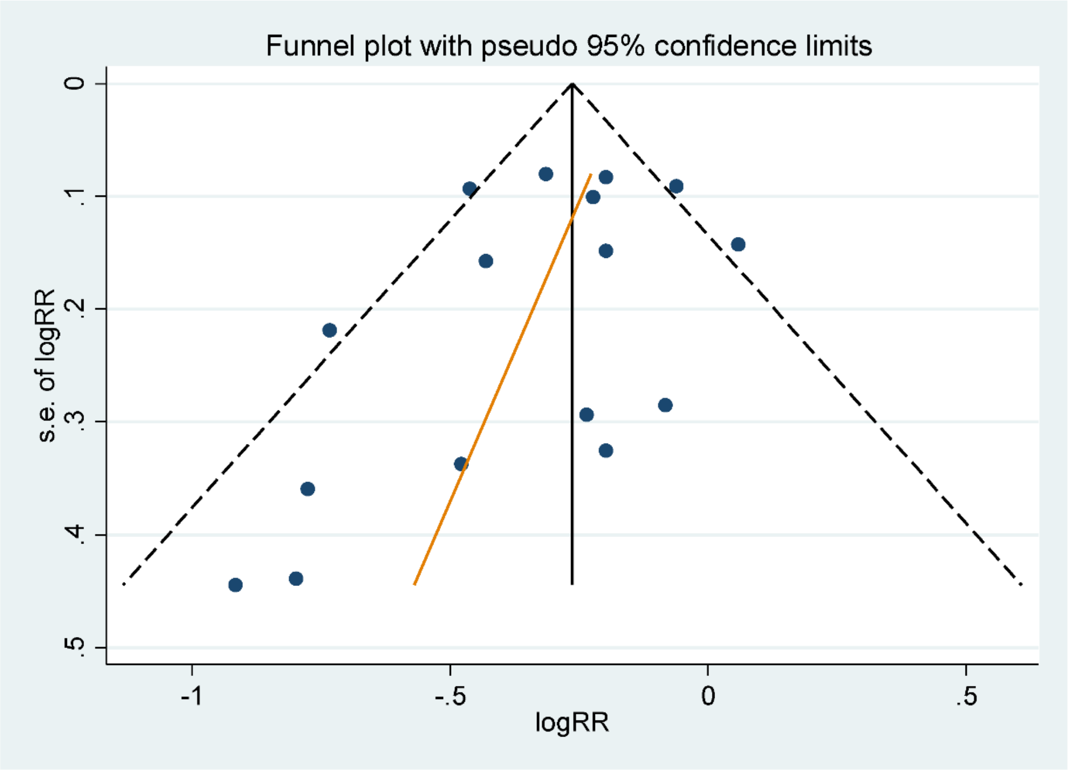


Figure S4: Funnel plot of the relative risk of 8 studies on FV consumption and COPD. Each dot represents a different study.

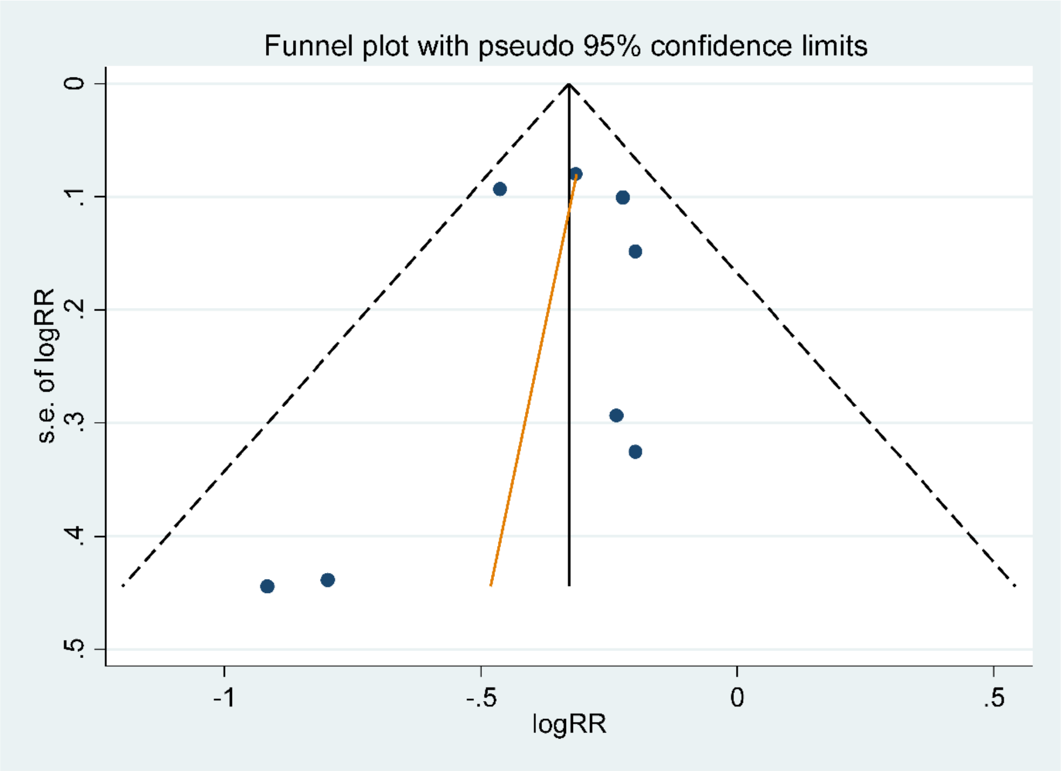


Figure S5: Funnel plot of the relative risk of 8 studies on fruit consumption and COPD. Each dot represents a different study.

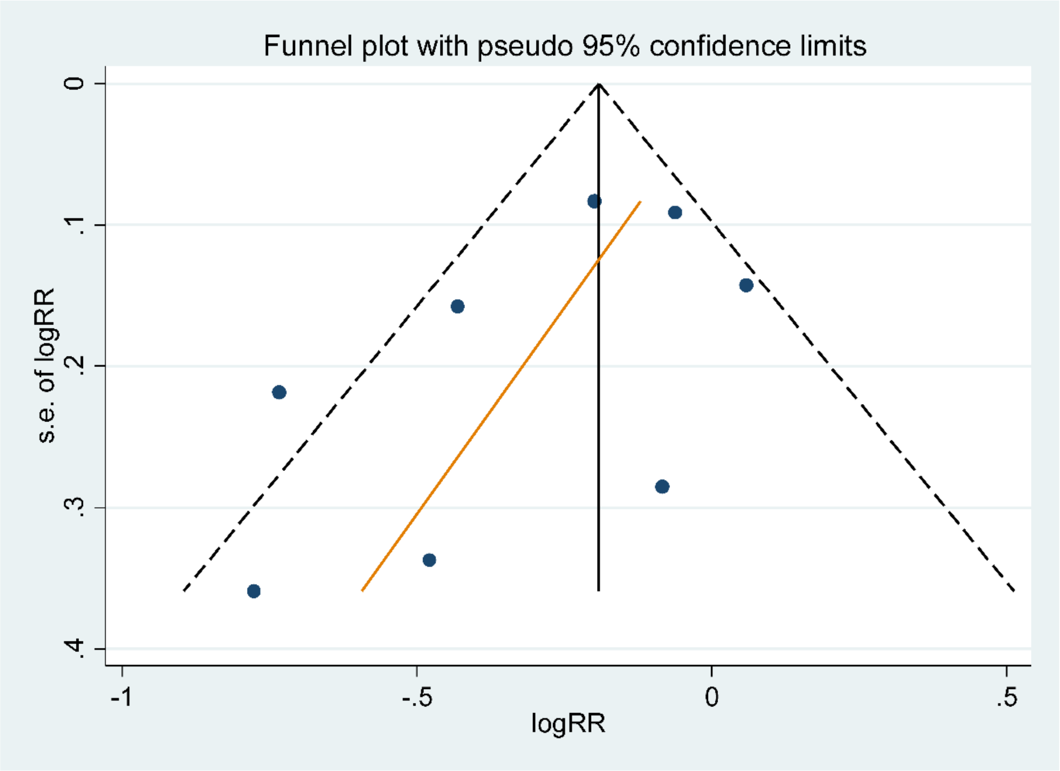


Figure S6: Funnel plot of the relative risk of 8 studies on vegetable consumption and COPD. Each dot represents a different study.

A Proposed Reporting Checklist for Authors, Editors, and Reviewers of Meta-analyses of Observational Studies.

| Checklist item | Reported on page # |
|---|---|
| Reporting of background should include | |
| 1. Problem definition | 2-3 |
| 2. Hypothesis statement | 2-3 |
| 3. Description of study outcome(s) | 2 |
| 4. Type of exposure or intervention used | 2 |
| 5. Type of study designs used | 2 |
| 6. Study population | 6-8 |
| Reporting of search strategy should include | |
| 7. Qualifications of searchers (eg, librarians and investigators) | 3 |
| 8. Search strategy, including time period included in the synthesis and keywords | 3-4 |
| 9. Effort to include all available studies, including contact with authors | 3-4 |
| 10. Databases and registries searched | 3 |
| 11. Search software used, name and version, including special features used (eg, explosion) | 3 |
| 12. Use of hand searching (eg, reference lists of obtained articles) | 3 |
| 13. List of citations located and those excluded, including justification | 4 |
| 14. Method of addressing articles published in languages other than English | 4 |
| 15. Method of handling abstracts and unpublished studies | 4 |
| 16. Description of any contact with authors | 4 |
| Reporting of methods should include | |
| 17. Description of relevance or appropriateness of studies assembled for assessing the hypothesis to be tested | 5 |
| 18. Rationale for the selection and coding of data (eg, sound clinical principles or convenience) | 6-7 |
| 19. Documentation of how data were classified and coded (eg, multiple raters, blinding, and interrater reliability) | 5 |
| 20. Assessment of confounding (eg, comparability of cases and controls in studies where appropriate) | 5 |
| 21. Assessment of study quality, including blinding of quality assessors; stratification or regression on possible predictors of study results | 4-5 |
| 22. Assessment of heterogeneity | 5-6 |
| 23. Description of statistical methods (eg, complete description of fixed or random effects models, justification of whether the chosen models account for predictors of study results, dose-response models, or cumulative meta-analysis) in | 5-6 |

| | |
|---|---|
| sufficient detail to be replicated | |
| 24. Provision of appropriate tables and graphics | 7,9-10,11,12,14,16 |
| Reporting of results should include | |
| 25. Graphic summarizing individual study estimates and overall estimate | 7-16 |
| 26. Table giving descriptive information for each study included | 9-10 |
| 27. Results of sensitivity testing (eg, subgroup analysis) | 12-16 |
| 28. Indication of statistical uncertainty of findings | None |
| Reporting of discussion should include | |
| 29. Quantitative assessment of bias (eg, publication bias) | 16 |
| 30. Justification for exclusion (eg, exclusion of non–English-language citations) | 4 |
| 31. Assessment of quality of included studies | 8 |
| Reporting of conclusions should include | |
| 32. Consideration of alternative explanations for observed results | 19-20 |
| 33. Generalization of the conclusions (ie, appropriate for the data presented and within the domain of the literature review) | 20 |
| 34. Guidelines for future research | 20 |
| 35. Disclosure of funding source | 20 |
